# Muscular and Systemic Correlates of Resistance Training-Induced Muscle Hypertrophy

**DOI:** 10.1371/journal.pone.0078636

**Published:** 2013-10-09

**Authors:** Cameron J. Mitchell, Tyler A. Churchward-Venne, Leeann Bellamy, Gianni Parise, Steven K. Baker, Stuart M. Phillips

**Affiliations:** 1 Exercise Metabolism Research Group, Department of Kinesiology, McMaster University, Hamilton, Ontario, Canada; 2 Department of Neurology, School of Medicine, McMaster University, Hamilton, Ontario, Canada; West Virginia University School of Medicine, United States of America

## Abstract

**Purpose:**

To determine relationships between post-exercise changes in systemic [testosterone, growth hormone (GH), insulin like grow factor 1 (IGF-1) and interleukin 6 (IL-6)], or intramuscular [skeletal muscle androgen receptor (AR) protein content and p70S6K phosphorylation status] factors in a moderately-sized cohort of young men exhibiting divergent resistance training-mediated muscle hypertrophy.

**Methods:**

Twenty three adult males completed 4 sessions•wk^-1^ of resistance training for 16 wk. Muscle biopsies were obtained before and after the training period and acutely 1 and 5 h after the first training session. Serum hormones and cytokines were measured immediately, 15, 30 and 60 minutes following the first and last training sessions of the study.

**Results:**

Mean fiber area increased by 20% (range: -7 to 80%; P<0.001). Protein content of the AR was unchanged with training (fold change = 1.17 ± 0.61; P=0.19); however, there was a significant correlation between the changes in AR content and fiber area (r=0.60, P=0.023). Phosphorylation of p70S6K was elevated 5 hours following exercise, which was correlated with gains in mean fiber area (r=0.54, P=0.007). There was no relationship between the magnitude of the pre- or post-training exercise-induced changes in free testosterone, GH, or IGF-1 concentration and muscle fiber hypertrophy; however, the magnitude of the post exercise IL-6 response was correlated with muscle hypertrophy (r=0.48, P=0.019).

**Conclusion:**

Post-exercise increases in circulating hormones are not related to hypertrophy following training. Exercise-induced changes in IL-6 correlated with hypertrophy, but the mechanism for the role of IL-6 in hypertrophy is not known. Acute increases, in p70S6K phosphorylation and changes in muscle AR protein content correlated with muscle hypertrophy implicating intramuscular rather than systemic processes in mediating hypertrophy.

## Introduction

It is well established that resistance training can lead to muscle hypertrophy [1], which appears to be the result of accumulated periods of post-exercise increases in muscle protein synthesis that exceed muscle protein breakdown, resulting in net protein accretion over time [2]. We have examined how differences in contractile paradigms affect skeletal muscle protein synthesis (MPS) [3–5]. Subsequently, we have studied the influence of some of these same variables in affecting skeletal muscle hypertrophy following prolonged training [6,7] with general agreement between short-term measurements of MPS and hypertrophy. While some studies have shown small effects of training variables such as rest periods between sets and relative training load on muscle hypertrophy [7,8], other studies have not [6,9]. A consistent observation is a high degree of heterogeneity in the hypertrophic response to resistance training [10,11]; the underlying causes of this variability in hypertrophic response are unclear.

As potential explanations for this hypertrophic heterogeneity changes in myogenic gene expression [12], microRNA abundance [13], and the capacity for myonuclear addition via satellite cells [14], have been examined. Other research has examined acute post-exercise changes in circulating factors proposed to be anabolic for muscle such as testosterone, growth hormone, and IGF-1 [15,16]. However, we have proposed, and provided evidence, that changes in these circulating factors after a single session of resistance exercise are unrelated to the magnitude of the MPS response or to muscular hypertrophy with resistance training [17–19]. In contrast, many state that the acute hormonal response to resistance exercise is an important driver of hypertrophy and have used a transient hormonal response to establish efficacy of interventions, including exercise and nutrition-based changes, to ascribe significance on a phenotypic and functional level; for reviews see [20,21]. Importantly, the anabolic action of testosterone in muscle is mediated via androgen receptors (AR), the mRNA for which has been shown to be up-regulated by resistance exercise [22], and that changes in its protein expression have been reported to correlate with the magnitude of muscle hypertrophy [23]; thus, we sought to ascertain whether changes in AR protein played a role in mediating hypertrophy.

The cytokine interleukin-6 (IL-6) has been implicated as a regulator of satellite cell function [24] and its release after resistance exercise correlates well with IL-6 measured in the muscle and in the blood [24]. Circulating IL-6 concentrations have also been taken as an indicator of resting inflammatory status [25]. Interestingly, in post-menopausal women a strong relationship between abdominal adiposity and IL-6 was negatively associated with changes in IL-6 and lean mass gain with resistance training [26]. It is unclear in young healthy men if the IL-6 response to resistance exercise is related to muscle hypertrophy after training.

In a retrospective analysis we reported that the acute response of various hormones (testosterone, GH, and IGF-1), often stated as being key anabolic drivers of hypertrophy [20,21] with resistance training, were unrelated to the hypertrophic response with resistance training [19]. This analysis [19] did, however, involve a nutritional manipulation, which could have obscured the true nature of the hormonal influence on hypertrophy. Thus, the purpose of the present study was to prospectively assess the relationship between the magnitude of the acute rise in circulating hormones – testosterone, GH, and IGF-1 – following resistance exercise early and late during a period (16 wk) of training and examine the relationship with muscle hypertrophy. We examined changes in muscle AR protein content and acute changes in p70S6K phosphorylation since they are proteins that have been shown to be related to the hypertrophic response in humans [23,27]. We also examined the relationship between the acute IL-6 response to resistance exercise and the magnitude of hypertrophy following training [24].

## Methods

### Subjects

Twenty three young healthy adult males (177 ± 8 cm, 84.1 ± 16.9 kg, 26.4 ± 4.4 kg/m^2^, 24 ± 3 yr) participated in the study. Subjects were recreationally active but had not participated in any resistance training for at least one year. All were deemed healthy based on responses to a standard medical screening questionnaire. Strength testing was conducted to determine voluntary isotonic strength defined by 1 repetition maximum (1RM) for the leg press and chest press exercise at the start and end of the training period. Subjects refrained from strenuous physical activity for 72 h before the testing sessions.

### Ethics Statement

The study was approved by the Hamilton Health Sciences Research Ethics Board and conformed to standards for the use of human participants in research as outlined in the 5th Declaration of Helsinki and with current Canadian Tri-council research agency guidelines for use of human participants in research (http://www.pre.ethics.gc.ca/eng/policy-politique/initiatives/tcps2-eptc2/Default/). Informed written consent was obtained from all participants prior to entering the study.

### Resistance Training Protocol

Subjects underwent 16 wk of progressive full body resistance training consisting of four training sessions per week. Each week consisted of two upper body and two lower body training sessions. The upper body exercises were chest press, shoulder press (Hammer Strength, Lake Forest, IL), lat pull down, rhomboid row (Atlantis, Laval, Quebec), bicep curl, and triceps extension (Hur, Kokkola, Finland). The lower body exercises were leg press (Maxam, Hamilton, Ontario), leg extension (Atlantis, Laval, Quebec), leg curl and calf press (Hur, Finland). The program consisted of 4 blocks of 4 weeks each. The first block consisted of 3 sets (2 sets for the first week) of 12 repetitions for each exercise and 60s rest between sets. Block two consisted of 3 sets (4 during the last week) of 10 with 90s rest between sets. The third block consisted of 4 sets (two sets for week 1) of 8 repetitions with 100s rest between sets. The last block consisted of 4 sets (3 sets for week 1) of 6 repetitions with 120s between sets. The last set for each exercise performed was performed to the point of momentary muscular failure. To maximize the potential for strength and lean mass gains as a result of training [28] subjects immediately consumed, after each training session and with breakfast on non-training days, a protein beverage containing 30 g of milk protein, 25.9 g of carbohydrates and 3.4 g of fat (Musahi P30, Notting Hill, Australia).

### Hormone/Cytokine Testing

After the strength testing, subjects rested for at least 96 h. Subjects reported to the lab for a resting blood sample and muscle biopsy of their vastus lateralis muscles. Subjects then underwent an acute bout of resistance exercise which consisted of leg press, leg curl, leg extension and calf press performed for 4 sets of 8 repetitions with 2 minutes rest between sets. At the end of the resistance exercise session, a blood sample was taken and the protein beverage described above was consumed. Blood samples were then taken 15, 30 and 60 minutes post exercise. Muscle biopsies (~100mg) were also taken 1 hour and 5 hours after the resistance exercise. The acute resistance exercise session were repeated after the training period, the 8RM load was set based on the subjects my recent 1RM test.

### Western Blotting

Muscle samples were homogenized on ice in buffer as previously described [29]. Protein concentrations were determined via BCA protein assay (Thermo Scientific, Rockford, IL, USA). Working samples of equal concentration were then prepared in Laemmli buffer [30] and equal amounts of protein (20 µg) were loaded onto 10% precast gels (BIO-RAD Mini-PROTEAN TGX Gels, Bio-Rad Laboratories, USA) for separation by electrophoresis. Proteins were then transferred to a polyvinylidene fluoride membrane, blocked in 5% skim milk (AR) or 5% Bovine serum albumin (p70S6K) for 1 h, and incubated overnight at 4°C in primary antibody against the AR receptor (1:2000, abcam, #ab3510) or phosphorylated p70S6K^Thr 389^. (1:1000 Santa Cruz Biotechnology # sc-11759) Membranes were then washed in TBST and incubated in secondary antibody for 1h at room temperature before protein detection with chemiluminescence (SuperSignalWest Dura Extended Duration Substrate, ThermoScientific, #34075) on a FluorChem SP Imaging system (Alpha Innotech, Santa Clara, CA, USA). Total AR and phosphorylated p70S6K protein was expressed relative to α-tubulin abundance (1:2000, Sigma-Alderich, St. Louis, MO, USA #T6074) and is presented as fold-change from pre- to post-training. Images were quantified by spot densitometry using ImageJ software (National Institute of Health, USA).

### Blood Analysis

*S*erum blood samples were collected into 4 ml evacuated tubes from a 22 gauge polyurethane catheter inserted into the antecubital vein while a 0.9% saline drip was used to keep the catheter patent. Serum was then left to clot at room temperature for one hour before being centrifuged at 4000rpm for 10 minutes. Blood plasma was then collected and frozen at -20°C until further analysis. Plasma samples were analyzed for serum growth hormone, free testosterone, IGF-1, IL-6, TNF-α, CRP and cortisol using solid-phase, two site chemiluminescence immunometric assays (Immulite; Intermedico, Holliston, MA). All intra-assay coefﬁcients of variation for these hormones were below 5% and all assays included external standards and daily quality controls. The hormone concentrations are uncorrected for changes in plasma volume since these are the concentrations to which potential target tissues would have been exposed.

### Muscle Fiber CSA

Muscle biopsies were obtained before and after the 16 wk training period from the vastus lateralis muscle using a 5-mm Bergstrӧm needle custom modified for manual suction under local anaesthesia (2% xylocaine). Muscle biopsy samples were embedded in optimal cutting temperature (OCT) and frozen in liquid cooled isopentane in preparation for sectioning and analysis via immunofluroescence. Muscle sections 7µm in thickness were cut placed on a glass slide and allowed to cool at room temperature for 30min before being fixed for 10min in 4% paraformaldehyde. Sections were then washed 3x5min in phosphate buffered saline (PBS) with Tween, and blocked for 1hr RT (in PBS containing 2% bovine serum albumin, 5% fetal bovine serum, 0.2% Triton x-100, 0.1% sodium azide, and 2% goat serum). Primary antibody incubation in Laminin (1:750, Abcam ab11575), MHCI (neat, DSHB), and MHCII (1:1000, Abcam ab91506) was completed for 2hr RT or overnight at 4°C. Secondary antibody detection included Laminin (Alexa Fluor 488 goat anti-rabbit, 1:500, Invitrogen A11008), MHCI (Alexa Fluor 488 goat anti-mouse, 1:500, Invitrogen A11029), and MHCII (Alexa Fluor goat anti-rabbit, 1:500, Invitrogen A21244), for 2hr at RT. Nuclei were revealed with DAPI (4',6-diamidino-2-phenylindole) (1:20000, Sigma D-8417), followed by cover slipping slides with fluorescent mounting media (DAKO S3023). Images were revealed with the Nikon Eclipse 90i microscope at 200x magnification and captured with a high-resolution QImaging fluorescent camera (Nikon, Tokyo, Japan). Fibre CSA was quantified manually in a blinded fashion using the Nikon NIS Elements AR 3.0 software on large scale images consisting of >100 fibres.

### Statistics

Pre to post training changes with a single mean were assessed using paired Student’s T-tests. Relationships between variables were assessed with Pearson’s product moment correlation. Changes in muscle fiber area were assessed with a two way (time by fiber type) repeated measures ANOVA. A stepwise multiple linear regression model was used to assess the contribution of various independent variables to the change in mean fiber area. Only those independent variables that were significantly correlated with changes in mean fiber area and had F probabilities less than 0.05 were entered into the model. The model with the highest proportion of variance, which met criteria above, is reported. Alpha was set at *P<0.05* and results are reported as means ± SD. All analyses were completed using SPSS version 20 (IBM, Armonk, New York).

## Results

Sixteen weeks of resistance training resulted in a 61% increase in isotonic strength as measured on the leg press, and a significant increases in both type I (18%; range -22 to 106%) and II muscle fiber area (23%; range -4 to 67%), with a significantly greater increase in type II fiber area (P=0.011).

**Figure 1:**
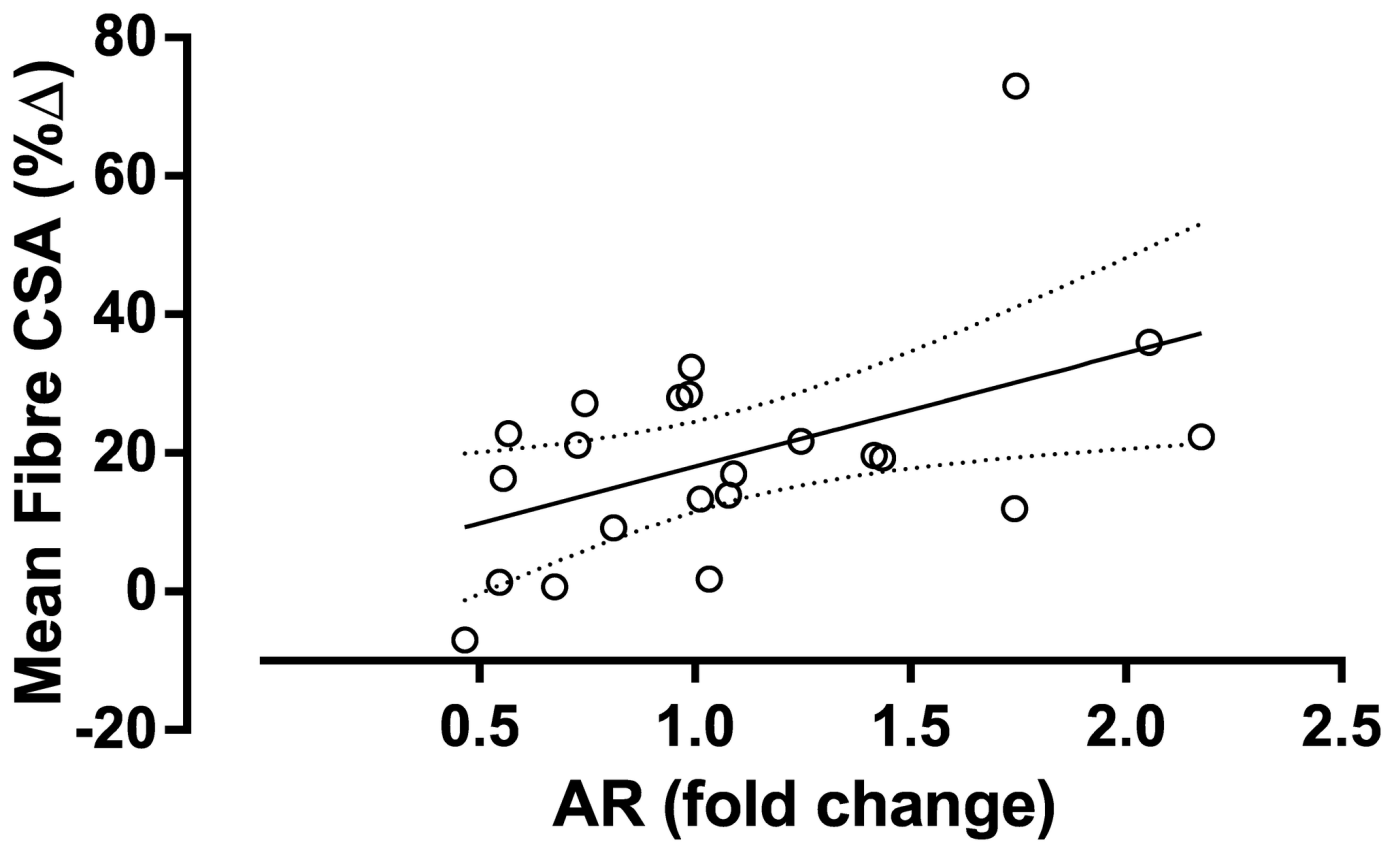
Correlation between the fold change in muscle AR protein content and changes in skeletal muscle fibre area following 16 weeks of resistance training. r = 0.60, P=0.003.

**Figure 2:**
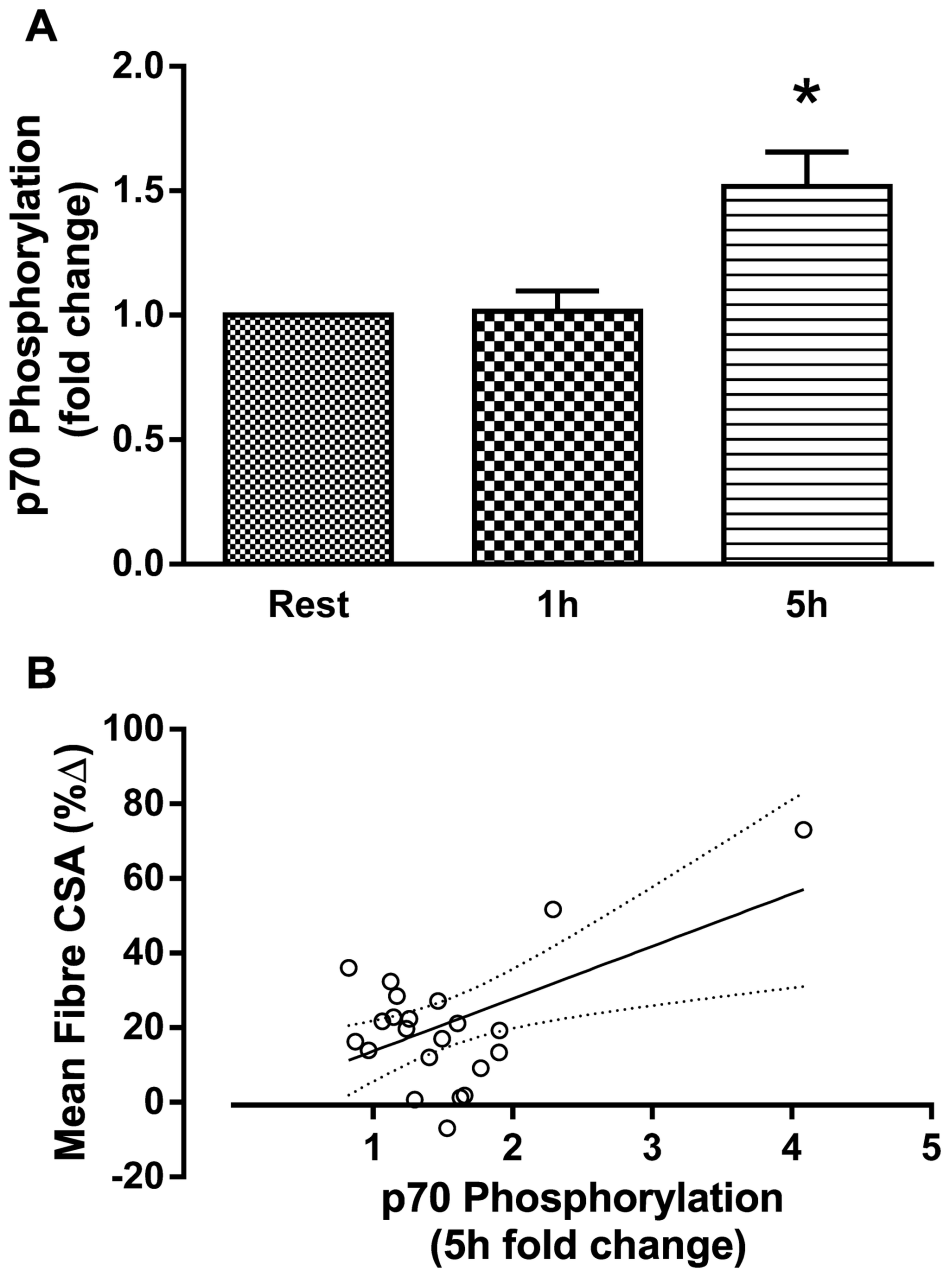
Phosphorylation of p70S6K following an acute bout of resistance exercise before 16 weeks of resistance training and the percentage change in skeletal muscle mean fibre area following the training * P < 0.05. A) Fold change in P70S6K phosphorylation. B) Correlation between 5h fold change in P70S6K phosphorylation and the percentage change in skeletal muscle mean fibre area following the training period. r = 0.54, P = 0.007.

**Figure 3:**
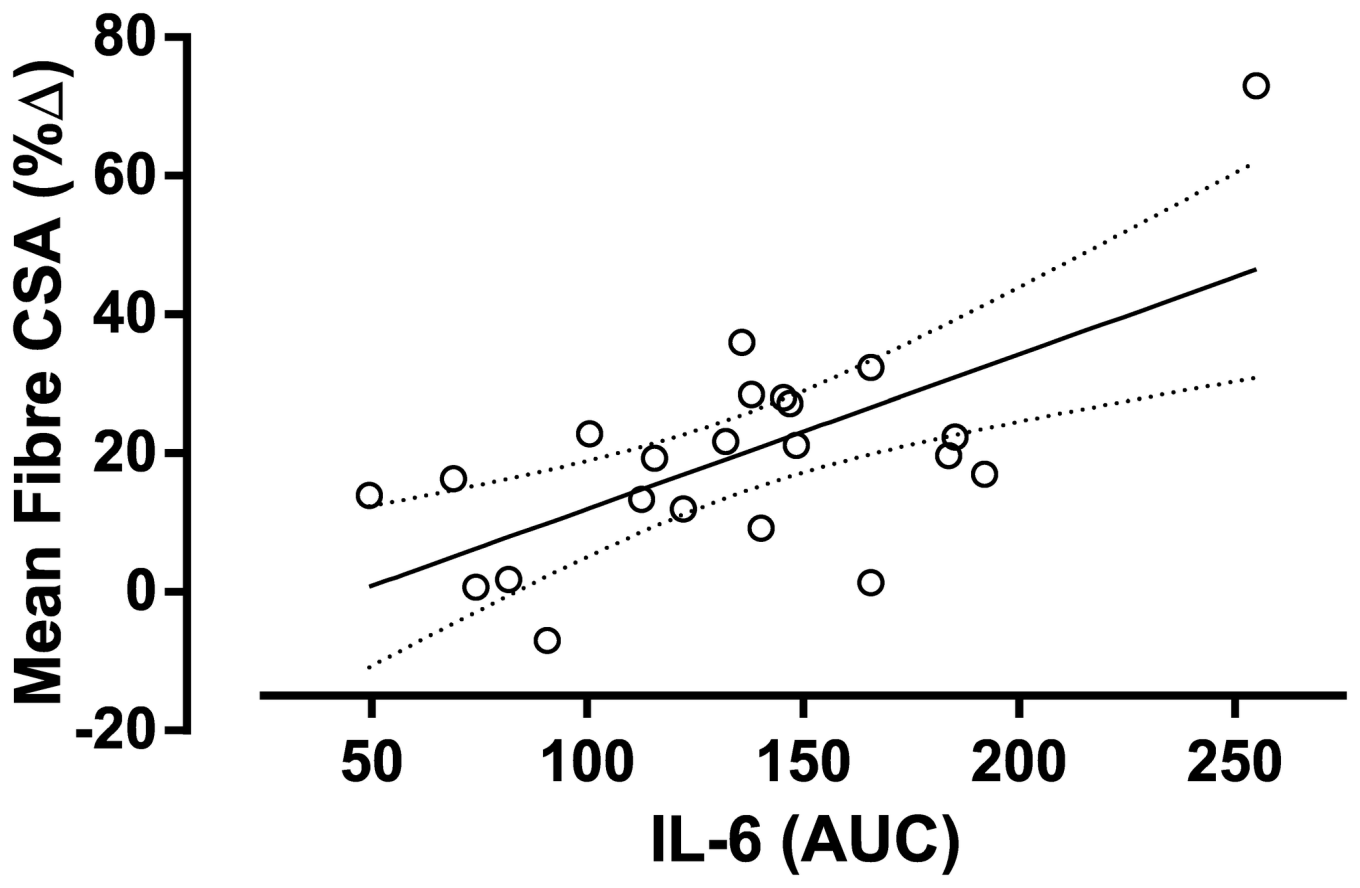
Correlation between the AUC of the acute IL-6 response to resistance exercise before 16 weeks of resistance training and the percentage change in skeletal muscle mean muscle fibre area following 16 weeks of resistance training. r=0.48, P = 0.019.

As a result of training there were significant reductions in the resting concentrations of free testosterone, GH, IGF-1and cortisol. There were no changes in the resting concentration of IL-6 or TNFα, however, there was an increase in the concentration of CRP (Table 1). The acute post-exercise AUC for free testosterone, GH, and IGF-1 decreased from pre- to post-training. The magnitude of the acute cortisol, IL-6, TNF-α and CRP responses were unaffected following 16 weeks of resistance training (Table 2). Mean muscle AR protein content was unchanged following the training period; however, there was a significant correlation between the change in AR content and the increase in mean fiber area (Figure 1). Phosphorylation of p70S6K was not increased above rest at 1 h following an acute bout of resistance exercise, but was significantly increased at 5 h post exercise (Figure 2a). Phosphorylation of p70S6K (fold change) at 5h was correlated with changes in muscle fiber area (Figure 2b) There were no relationships between free testosterone, GH, and IGF-1 post exercise AUC responses measured before (Table 3) or after (free testosterone: r=0.02, *P* = 0.932, GH: r=0.37, *P* = 0.079, IGF-1: r=-0.25, *P* = 0.249) the 16 week training period and changes in mean muscle fiber area CSA (Table 3). There was a significant relationship between the acute response of IL-6 both pre- (Figure 3) and post-training (r=0.47, *P* = 0.023) and increases in mean muscle fibre CSA. A stepwise multiple linear regression model revealed that only two variables accounted for a significant proportion of variance related to the training-induced change in mean fiber area: change in AR protein content and the magnitude of p70S6K phosphorylation at 5h (Table 4). The R^2^ of the model after adjustment for multiple variables was 0.46. Although IL-6 AUC was significantly correlated with changes in mean fiber area it was not included because the F probability when included in the model was greater than 0.05.

**Table 1:** Pre- and post-training resting hormone and cytokine concentrations, muscle fibre cross-sectional area, and strength.

	Pre	Post	P
Free Testosterone (pM)	150 ± 9	144 ± 9	0.000
GH (ug/L)	1.6 ± 0.5	1.4 ± 0.4	0.009
IGF-1 (nM)	38.4 ± 3.9	37.0 ± 4.6	0.005
Cortisol (nM)	593 ± 84	543 ± 74	0.000
IL-6 (pg/mL)	2.2 ± 0.6	2.1 ± 0.7	0.122
TNF-alpha (pg/mL)	0.87 ± 0.34	0.78 ± 0.26	0.186
CRP (nM)	102 ± 9	110 ± 19	0.007
Mean CSA (µm^2^)	5928 ± 1651	7013 ± 1471	0.000
Type II CSA (µm^2^)	6284 ± 1869	7542 ±1736	0.000
Type I CSA (µm^2^)	5355 ± 1553	6098 ±1486	0.001
Leg Press 1RM (kg)	236 ± 70	380 ±73	0.000
Androgen Receptor (fold change)	1	1.17 ± 0.61	0.186

GH – growth hormone, IGF-1 – insulin-like growth factor-1, TNF-α – tumour necrosis factor-α, CRP – C-reactive protein, CSA – cross sectional area, 1RM – single repetition maximal strength.

**Table 2:** Pre- and post-training area under the curve of acute hormonal and cytokine responses to a single bout of resistance exercise.

	Pre	Post	P
Free Testosterone	7557 ± 1414	7190 ± 1417	0.000
GH	443 ± 158	420 ± 149	0.000
IGF-1	398 ± 478	335 ± 499	0.043
Cortisol	5103 ± 9157	4613 ± 8019	0.142
IL-6	131 ± 48	131 ± 48	0.261
TNF-alpha	17± 17	18 ± 19	0.119
CRP	572 ± 771	580 ± 1424	0.968

GH – growth hormone, IGF-1 – insulin-like growth factor-1, TNF-α – tumour necrosis factor-α, CRP – C-reactive protein. Area under the curve was calculated based on the serum concentrations immediately after exercise and, 15, 30 and 60 min following exercise with baseline (resting) values subtracted. AUC values presented in arbitrary units.

**Table 3:** Pearson correlations between pre-training hormone, cytokine, and androgen receptor (AR) response and changes in muscle fibre cross-sectional area (CSA) following training.

Variable		Change in Mean CSA	Change in Type II CSA	Change in Type I CSA
Fold change AR	r	0.60	0.60	0.47
	P	**0.003**	**0.002**	**0.023**
Free Testosterone AUC	r	0.06	0.07	0.10
	P	0.771	0.760	0.639
IL-6 AUC	r	0.48	0.42	0.51
	P	**0.019**	**0.047**	**0.013**
GH AUC	r	0.39	0.40	0.38
	P	0.069	0.061	0.073
IGF-1 AUC	r	-0.30	-0.27	-0.23
	P	0.165	0.219	0.292
Cortisol AUC	r	-0.02	0.00	-0.09
	P	0.919	0.990	0.670
TNF-alpha AUC	r	0.02	-0.05	0.10
	P	0.919	0.812	0.652
CRP AUC	r	0.18	0.13	0.06
	P	0.420	0.567	0.784

AR – androgen receptor, IL-6 – interleuking-6, GH – growth hormone, IGF-1 – insulin-like growth factor-1, TNF-α – tumour necrosis factor-α, CRP – C-reactive protein, CSA – cross sectional area, 1RM – single repetition maximal strength

**Table 4:** Stepwise Multiple Regression Model.

Model	Unstandardized Coefficients			Standardized Coefficients		P
	B	SE		β	t	
Constant	-10.6	7.5			-1.4	0.174
AR	13.4	4.6		.48	2.9	0.008
P70S6k	10.4	4.2		.40	2.4	0.023

AR − Dependant variable was the percentage change in mean fiber area

## Discussion

As with many phenotypic changes with exercise training muscular hypertrophy in response to resistance training shows a high degree of variability, the source of which is unknown [10,11]. We discovered that the increase in AR protein content with resistance training, the magnitude of acute increase in p70S6K phosphorylation, and the response of IL-6 to an acute bout of resistance exercise were significantly correlated with the magnitude of muscle fiber hypertrophy in a moderate-sized cohort of young men following 16 weeks of resistance. Using multiple regression analysis we found that only changes in AR content and phosphorylation of p70S6K were significant variables in a model that accounted for 46% of the variance in the hypertrophic response. Our findings also corroborate previous work from our lab showing no relationship between the acute increase in circulating free- testosterone, IGF-1, or GH and the magnitude of muscle hypertrophy following training [19].

The exact role of the AR in muscle hypertrophy has yet to be elucidated. The abundance of the muscle AR mRNA does not increase acutely following resistance exercise but does tend to increase 48h following an acute bout of resistance exercise [22,23]. This pattern of AR mRNA up regulation persists for at least three training sessions but does not seem to be preserved with longer term training [31]. Our study corroborates previous findings that mean AR protein expression was not increased following resistance training; however, the response had marked heterogeneity, with some subjects showing a marked (1.5 - 2.5 fold) increase in AR protein content [23]. Despite no statistically significant change in AR receptor protein content, there was a correlation between AR protein content with fibre hypertrophy. Our results suggest that changes in AR content may be part of a muscle-specific response present to a greater degree in responders and responsible for some (~25%) of the variation in muscle fibre hypertrophy [32].

The protein kinase B (Akt)-mammalian target of rapamycin (mTOR) pathway is thought to be critically important in regulating muscle protein synthesis and hypertrophy. Although the mTOR complex is an important regulator of protein synthesis, simple phosphorylation of mTOR has never been shown to correlate with muscle protein synthesis or hypertrophy, thus it was not measured in this study. The protein kinase p70S6K is a target of mTOR and its acute phosphorylation following resistance exercise has been reported to correlate with muscle hypertrophy following training [27,33]; however, not all studies have found a relationship [6,34]. We saw no increases in p70S6K phosphorylation 1h after exercise, however, phosphorylation was elevated 5h post-exercise. The 5h phosphorylation was correlated with muscle hypertrophy suggesting that individuals who showed a later (5h) phosphorylation of p70S6K may exhibit greater hypertrophy. In this study basal fasting phosphorylation status was compared to the phosphorylation status of each target after both an acute exercise bout and the ingestion of a drink containing 30g of protein. It is possible that the observed results were partly due to the effects of nutation alone [35]. We chose to examine the acute phosphorylation of proteins to the response to combined exercise and nutrition because the provision of post exercise protein results in greater hypertrophy following training [28] and subjects were given an identical protein supplement after every workout.

The lack of correlation between free testosterone and muscle hypertrophy is in agreement with previous work from our laboratory [19,36]. Supraphysiological doses of testosterone dramatically enhance resistance training-induced muscle hypertrophy [37]; in contrast, the current, and previous [19,36], findings of no relationship between the acute rise in testosterone and muscle hypertrophy could be explained in one of two ways. First, other local factors are far more important in regulating muscle hypertrophy, and testosterone is thus merely a permissive hormone within its normal physiological range and only has affects during hypo- or hyper-testosteronemic states. The other possible explanation is that the intramuscular concentration of testosterone, where it is more active and bound to its receptor protein, is poorly related to its concentration in circulation. Thus, measurement of circulating testosterone concentrations following exercise is not a marker for, or in any way related to, skeletal muscle hypertrophy; however, intramuscular testosterone, or testosterone-AR complex formation, may be important regulators of protein synthesis and muscle hypertrophy.

IGF-1 is thought to be a potential regulator of muscle hypertrophy acting directly through the Akt/mTOR pathway [38] as well as co-localizing with satellite cells following resistance exercise [39]. It is proposed that local autocrine or paracrine IGF-1 plays an important role in hypertrophy [40]. There is no evidence, however, that the hepatic-derived IGF-1, mediated through release via the GH-IGF-1 axis, and measured in circulation is related to levels in the muscle. In fact, a recent study by Nindl and colleagues showed that there was no relationship between IGF-1 measured in the blood and IGF-1 protein content within the muscle [41]. The lack of correlation between muscle hypertrophy and IGF-1 measured in the blood observed in this study is not surprising in light of the work by Nindl et al. showing no relationship between IGF-1 protein in the muscle or interstitial space and IGF-1 in the blood [41].

We observed a correlation between the magnitude of the IL-6 response post-exercise and fibre hypertrophy. We also found that pre-training resting IL-6 was inversely correlated with fibre hypertrophy. IL-6 is associated with both muscle protein breakdown and JAK/STAT signalling in satellite cells, which are linked to muscle hypertrophy [24]. High resting levels of IL-6 are seen with aging, systemic inflammation and are associated with greater levels of visceral adiposity [25,42,43]. Conversely IL-6 is released from muscle following exercise and although there is a pronounced rise in the systemic circulation, the main post-exercise effects are likely to be autocrine or paracrine within the muscle [24,44]. Despite the observed correlations of the acute serum IL-6 response and hypertrophy we report here, when added to a multiple regression model the influence of circulating IL-6 is minor by comparison to measures of muscle-specific factors: AR and p70S6K. The serum IL-6 response immediately following exercise may be a ‘diluted’ version of the response within the exercising muscle; thus, measurement of IL-6 in the muscle itself would be preferable to blood concentrations.

The multiple linear regression model showed that the significant model terms for the magnitude of the increase mean muscle fiber area were the change in AR protein content (Beta=0.480) and the degree of p70S6K phosphorylation 5 hours after an acute bout of resistance exercise (Beta=0.404). Although the acute IL-6 response was correlated with changes in mean fiber area it did not add to the variance in hypertrophy explained by the model and so was not included. Our model shows that intrinsic factors within the muscle explain more unique variance in the hypertrophic response compared with circulating factors. The model also showed that p70S6K phosphorylation and AR protein content explained unique variance in mean fiber area gains and thus they are likely acting though different pathways or they represent acute and chronic effects in a linked pathway since p70S6K phosphorylation was an acute measure whereas AR receptor changes represent a change over the 16 week training period.

During the study period subjects consumed a drink containing 30 g of protein after each training session and with breakfast on non-training days. Subjects refrained from consuming any additional supplements and we told to eat the foods that made up their normal diet *ad libitum*. It seems unlikely that total energy or protein intake were substantial contributors to between subject variability in muscle hypertrophy following the resistance training; previous work has shown no difference in self-reported dietary intake between extreme- and non- responders to resistance training [45]. It would be ideal for future studies to tightly control dietary intake by providing subjects with food to eliminate any potential effect of dietary intake, however, this would be logistically difficult, expensive, and, we propose, unlikely to allow a ‘non-responder’ to become a ‘responder’ in terms of hypertrophy.

In the current study we chose to measure muscle hypertrophy with the analysis of muscle fiber area. The pattern of results was identical for both type I and type II muscle fibers for this reason we used mean fiber area for our multiple regression model. We were not able to differentiate between fiber sub types (IIa or IIx) or hybrid fiber which is a small limitation of this study. A whole muscle homogenate was used for the AR and p70S6K analysis to further the mechanistic understanding of fiber type specific hypertrophy future studies could compare homogenates isolated from specific fiber type with hypertrophy in only fibers of the same type.

Muscle hypertrophy is mainly, if not entirely, a muscle-drive process that is regulated primarily by mechanisms intrinsic to the muscle rather than by systemic factors, which appear permissive until extreme hyper- or hypo-states are present. Changes in AR content and p70S6K phosphorylation, or as we have reported previously expression of the muscle-specific microRNA (miRNA) mi451 [13], within the muscle account for more variability in training induced muscle hypertrophy as compared to measuring multiple putative anabolic hormones (T, GH, IGF-1) in systemic circulation. While we observed a significant correlation between circulating IL-6 and hypertrophy when this was added to a model that contained muscle protein measurements IL-6 did not account for any additional variance in the hypertrophic response. The ability to predict a phenotypic response to training based on variables measured following a single session of resistance exercise has some support when measuring muscle-specific variables [13,14]; however, future studies should focus on quantifying factors intrinsic to the muscle or systemic molecular signatures, not of single or multiple hormones, that could act as a surrogates for relevant muscle intrinsic drivers of hypertrophy. Markers intrinsic to skeletal muscle such as AR, p70S6K, miRNA, or transcriptomic profiles will explain much more variance in muscle hypertrophy following resistance training than systemic factors such as T, GH, cortisol or IGF-1.
